# The 2018 Classification of Periodontitis: Challenges from Clinical Perspective

**DOI:** 10.3390/dj13080361

**Published:** 2025-08-08

**Authors:** Marek Chmielewski, Andrea Pilloni, Alessandro Cuozzo, Giuseppe D’Albis, Gerarda D’Elia, Piero Papi, Lorenzo Marini

**Affiliations:** 1 Private Practice, 81-881 Sopot, Poland; machmielewski@proton.me; 2 Section of Perioontics, Department of Oral and Maxillofacial Sciences, Sapienza University of Rome, 00161 Rome, Italy; delia.1636252@studenti.uniroma1.it (G.D.); piero.papi@uniroma1.it (P.P.); lorenzo.marini@uniroma1.it (L.M.); 3 Periodontology Unit, Department of Neuroscience, Reproductive and Odontostomatological Sciences, University of Naples Federico II, 80131 Naples, Italy; alessandro.cuozzo@unina.it; 4 Department of Interdisciplinary Medicine, University of Bari “Aldo Moro”, 70121 Bari, Italy; giuseppe.dalbis@uniba.it

**Keywords:** classification, data accuracy, diagnosis, periodontal disease, periodontitis, reproducibility of results

## Abstract

**Objectives**: The objective of this narrative review was to evaluate the clinical challenges encountered in the application of the 2018 AAP/EFP Classification of Periodontitis. **Methods**: Electronic and manual searches were conducted to identify studies reporting diagnostic accuracy and inter- and intra-examiner agreement when using the 2018 Classification, both with and without the aid of implementation tools. **Results**: Eleven studies were included, encompassing a total of 459 clinical cases. Overall, 852 examiners participated, with 31 gold-standard examiners providing the reference diagnoses. General dentists often exhibited lower diagnostic accuracy and consistency compared to students and periodontal experts. Diagnostic challenges were observed in staging, particularly distinguishing between Stage III/IV and gingivitis/Stage I. Grading reliability was reduced in the absence of longitudinal data and high-level modifying factors. This review also explored difficulties in reporting ‘hopeless teeth’ and assigning the extent of periodontitis. Education, training, and implementation tools substantially improved diagnostic accuracy and consistency, increasing the agreement with reference diagnosis and strengthening inter- and intra-examiner agreement. **Conclusions**: The application of the 2018 Classification showed notable variability in diagnostic accuracy and inter- and intra-examiner agreement. Improving clinician experience and training and incorporating diagnostic aids and emerging AI technologies has the potential to enhance diagnostic accuracy and consistency, which are essential for precisely estimating periodontitis prevalence, interpreting research findings, and determining prognosis and treatment needs.

## 1. Introduction

The 2018 AAP/EFP Classification of Periodontal and Peri-implant Diseases and Conditions released by Caton et al. [1] is approaching its seventh anniversary, offering a significant update over the previous classification system [2]. In particular, the 1999 Classification differentiated forms of periodontitis using categories such as ‘aggressive’ and ‘chronic’, which were often misleading and lacked a biological basis, thereby complicating accurate patient diagnosis [3]. Furthermore, there was a need for a classification system that went beyond simply assessing disease severity and captured the multiple dimensions of an individual’s condition. The staging and grading system proposed by the 2018 Classification, which also considers modifiers such as smoking and glycated hemoglobin levels, facilitates a more personalized diagnosis and treatment plan tailored to each patient’s needs [3].

Following the 2018 Classification introduction, decision-making algorithms and dedicated software have been developed to support clinicians during chair-side evaluations, streamlining and enhancing the diagnostic process [4,5]. Building on these technological advances, recent progress in artificial intelligence and deep learning offers practitioners powerful tools for accurate radiographic bone loss analysis and early assessment of periodontal conditions [6,7,8]. These innovations hold significant potential to improve the early detection and management of periodontal disease, ultimately leading to better patient outcomes through timely intervention.

Reliable case definitions and diagnosis are essential for successfully estimating the prevalence of periodontitis, interpreting research findings, and determining the patient prognosis and a treatment plan. Importantly, the classification system is used not only by periodontists but also by general dentists and dental students at various stages of their education. Therefore, it is not surprising that numerous studies have been conducted to assess diagnostic consistency and accuracy within the staging and grading system.

This narrative review aims to summarize the inter- and intra-examiner agreement associated with the application of the 2018 Classification of Periodontitis, while also highlighting the challenges faced by clinicians with varying levels of periodontal expertise and training, including issues related to the use of implementation tools. The narrative approach was intentionally selected to provide a flexible and interpretive framework, allowing for a critical analysis of a broader and more heterogeneous body of evidence.

## 2. Materials and Methods

The present narrative review focused on studies assessing intra- and inter-examiner agreement in the use of the 2018 Classification of Periodontitis, both with and without the aid of implementation tools.

### 2.1. Information Sources

Database searching comprised papers published in PubMed and Embase from 2018 to February 2025. The search strategy was as follows: (Periodontitis OR periodontal) AND (classification OR diagnosis OR case definition OR staging OR grading) AND (EFP OR “European Federation of Periodontology” OR AAP OR “American Academy of Periodontology” OR “2017” OR “2018” OR Workshop) AND (accuracy OR consistency OR agreement OR reliability OR reproducibility).

A manual search was also performed, including targeted searches in key journals such as *Periodontology 2000*, *Journal of Dental Research*, *Journal of Clinical Periodontology*, *Journal of Periodontology*, *Journal of Periodontal Research*, *Clinical Oral Investigations*, and *Clinical Advances in Periodontics*. Peer-reviewed articles, reports, and book chapters published up to February 2018 were screened. Additionally, relevant narrative and systematic reviews were examined to identify suitable references.

### 2.2. Eligibility Criteria

Studies were included in this narrative review if they met the following criteria: (i) they evaluated the application of the 2018 Classification of Periodontitis, with or without the use of implementation tools; (ii) they involved participants such as specialist periodontists, general dentists, dental hygienists, dental therapists, or undergraduate/postgraduate dental students; (iii) they reported inter- or intra-examiner agreement outcomes, including percentage agreement, kappa statistics, or intraclass correlation coefficients; (iv) they were published in English; and (v) they were available in full-text.

The following were excluded from this narrative review: studies in which the periodontal cases assessed were diagnosed with periodontitis associated with systemic diseases/conditions or specific syndromes, studies focusing on the Classification of Peri-Implant Diseases, as well as pre-clinical, animal, or laboratory studies.

### 2.3. Screening and Data Extraction

Study selection was carried out independently by two reviewers (M.C. and G.D.E.) in two stages. First, titles and abstracts were screened to identify studies that met the inclusion criteria. Second, the full texts of potentially relevant articles were reviewed for eligibility. In cases of disagreement between the reviewers, consensus was sought through discussion. If consensus could not be reached, a third reviewer (A.P.) acted as an arbitrator and made the final decision regarding inclusion.

The following data were extracted: setting (country); number and education level of examiners; presence of a gold-standard diagnosis; inclusion of additional lectures on the 2018 Classification; use of implementation tools; number of periodontal cases assessed; accuracy of periodontal status diagnosis compared to the gold standard; inter-examiner agreement; and intra-examiner agreement.

## 3. Results

### 3.1. Study Characteristics

Studies from the literature addressing diagnostic accuracy, as well as inter- and intra-examiner agreement, along with their main features, are summarized in Table 1.

A total of 459 cases were diagnosed according to the 2018 Classification across the reviewed studies, with the majority from Raza et al. (336 cases) and the fewest in Bumm et al. (2 cases).

Case presentations mainly included medical/dental history, intraoral photographs, periodontal charts, and full-mouth X-rays. Only Bumm et al. [15] provided panoramic radiographs, while Pakdeesettakul et al. [14] did not specify radiograph types. Documentation was consistently sufficient for accurate evaluation by examiners.

Four studies [5,9,16,18] covered the full periodontitis spectrum (Stages I–IV), with some—including Abou-Array et al. [12], Pakdeesetkul et al., [14] and Raza et al. [17]—adding periodontal health and/or gingivitis cases for diagnostic complexity. Ravidà [10] and Oh [11] excluded Stage I cases, while Bumm [15] and Abrahamian [13] focused narrowly on advanced stages (III–IV), aiming to test diagnostic precision in advanced cases.

Across all studies, 31 gold-standard examiners provided a reference diagnoses. In nine studies, these diagnoses served as benchmarks for participant evaluations. The largest panels were seen in Abrahamian et al. (seven experts) [13] and Pakdeesettakul et al. (six experts) [14]; Alshehari et al. [18] and Marini et al. [5,9] relied on a single expert. Notably, Marini et al. and Ravidà et al. [5,9,10] involved experts who had contributed to developing the 2018 Classification, enhancing the authority of their gold-standard diagnoses.

A total of 852 examiners participated across the 11 studies, ranging from 1 [17] to 174 [13]. Examiners varied widely in experience, from periodontal experts to undergraduate students unfamiliar with the 2018 Classification. Several studies [9,13,14] compared expert and student responses against gold standards to assess the diagnostic accuracy and consistency of the 2018 system. Other studies [11,12,15,16,18] involved only students or postgraduates, while Ravidà et al. and Raza et al. [10,17] focused solely on expert examiners.

Five studies implemented preparatory training for examiners before diagnostic evaluation. Among these, Marini et al. [9] provided clear instructions and targeted training. Roshdy Abdelrasoul et al. [16] used a two-step calibration (seminar + test) to align examiner understanding, while Abou-Array et al. and Bumm et al. [12,15] offered dedicated lectures. Alshehari et al. [18] relied on a university course during the third year for examiner preparation. Diagnostic aids, such as decision-making algorithms [13,15] or simplified flowcharts [14], were tested in three studies, with the results showing improved diagnostic confidence and consistency.

### 3.2. Agreement with Gold-Standard Diagnosis

All studies assessed examiner accuracy using a gold-standard reference, with the results varying widely based on the examiner background, educational intervention, and case definition component (stage, grade, extent). Marini et al. [9] provided a comprehensive comparison across three examiner groups—undergraduates, general dentists, and periodontal experts—showing that although periodontal experts achieved the highest accuracy in staging (82.0%), undergraduates outperformed in grading (74.4%) and extent evaluation (87.6%). Surprisingly, undergraduates also achieved the highest overall diagnostic accuracy (53.6%), exceeding both experts (50.4%) and general dentists (37.6%). This suggests that structured academic training may be more effective than clinical experience alone in using the 2018 Classification system. Similarly, Ravidà et al. [10] reported variable accuracy among periodontal experts, with the lowest agreement observed in staging (76.6%) and the highest in extent assessment (84.8%). Abrahamian et al. [13] found that faculty achieved the highest staging accuracy (71.4%), specialist clinicians reported the highest accuracy of grade identification (86.1%), while postgraduates led in extent evaluation (77.7%). Across other studies, educational interventions also improved accuracy. Roshdy Abdelrasoul et al. [16] demonstrated that accuracy increased from 63.8% to 75.6% after enhanced teaching and case-based discussion, and Pakdeesettakul et al. [14] showed a small improvement with the use of diagnostic flowcharts. In these studies, grading and extent evaluation tended to yield higher accuracy than staging. In the study of Bumm et al. [15], logistic regression revealed the predictive value of experience (OR = 4.13) and algorithm use (OR = 11.90), highlighting the importance of structured support tools alongside clinical exposure. Table 2 reports the diagnostic agreement with gold-standard diagnosis.

### 3.3. Inter-Examiner Agreement

Six studies evaluated inter-examiner agreement in periodontal diagnosis using either the kappa coefficient or percentage agreement. Overall, moderate agreement was commonly reported. Marini et al. [9] showed that undergraduate examiners achieved the highest agreement, periodontal experts showed moderate-to-fair agreement, while general dentists exhibited lower consistency, especially for extent. Ravidà et al. [10] found moderate agreement for all categories: stage (k = 0.49), grade (k = 0.50), extent (k = 0.51), and overall diagnosis (k = 0.479). Abrahamian et al. [13] used percentage agreement, reporting the highest agreement for grade (82.4%), followed by extent (75.5%) and the lowest for stage (68.7%). Roshdy Abdelrasoul et al. [16] observed improvement in agreement after examiner calibration, from fair (pre-test k = 0.215) to moderate (post-test k = 0.427). Oh et al. [11] showed that periodontal examiners had moderate agreement (k = 0.41), while non-periodontal examiners had only fair agreement (k = 0.28), with overall agreement remaining fair (k = 0.34). Table 3 presents the data on inter-examiner agreement among the examiners.

### 3.4. Intra-Examiner Agreement

Six studies [9,11,12,13,14,15] evaluated intra-examiner agreement in periodontal diagnosis using either the kappa coefficient, intraclass correlation coefficient (ICC), or percentage agreement. Marini et al. [9] reported the highest levels of intra-examiner reliability across all examiner groups and diagnostic parameters. Undergraduate examiners achieved near-perfect agreement, with ICC values of 0.95 (stage), 0.88 (grade), and 0.98 (extent). Periodontal experts also demonstrated high agreement (ICC = 0.82–0.88), while general dentists showed substantial intra-examiner reliability for stage (ICC = 0.92) and grade (ICC = 0.86). These results suggest consistently high repeatability among both less experienced and expert clinicians when calibrated. Abrahamian et al. [13] observed similarly strong intra-examiner reliability, with kappa values of 0.71 (stage), 0.85 (grade), and 0.52 (extent), and corresponding percentage agreements of 82.3%, 91.4%, and 83%. Abou-Array et al. [12] found moderate intra-examiner agreement among postgraduate students (k = 0.55), while lower agreement levels were observed in orthodontic postgraduates (k = 0.30), fourth-year students (k = 0.26), and second-year students (k = 0.24), highlighting the influence of clinical experience and training. Oh et al. [11] reported overall intra-examiner agreement as fair (k = 0.34), suggesting lower repeatability in diagnostic assessments among the mixed examiner group studied. The intra-examiner agreement values are depicted in Table 3.

### 3.5. Identified Factors Affecting Stage and Grade Diagnosis Accuracy or Consitency

Across the studies, various factors were identified as influencing the accuracy or consistency of diagnosis and case definition, with the examiner experience and training, diagnostic methodology, and case complexity emerging as central themes (Table 4).

## 4. Discussion

Adopting a new classification system takes time, as it involves adjusting to a different conceptual approach. It is essential that such classifications are exhaustive, clear, simple to be used, and consistently reproducible. In fact, discrepancies in case definitions can greatly influence the estimated prevalence of periodontitis. These inconsistencies may also impact the findings and associations reported in studies. Additionally, there is a potential risk of either overestimating or underestimating the actual need for periodontal therapy [9,19].

This narrative review evaluated the current evidence on the clinical application of the 2018 Classification of Periodontitis. The findings provide valuable insights into the classification system’s performance, particularly regarding diagnostic accuracy and consistency. The following sections discuss the key themes that emerged from the reviewed studies.

### 4.1. Diagnostic Performance and Examiner Background

One of the most striking findings across the reviewed studies is the unexpectedly poor performance of general dentists compared to undergraduate students [9]. Given their broader clinical experience, this raises concern, particularly since general dentists serve as the first line of detection and referral in periodontal care. Several studies, including Oh et al. [11], reported that 79% of general dentists were either unaware of or not applying the 2018 Classification, whereas 74% of periodontists routinely used it. This knowledge gap may partly explain their reduced diagnostic accuracy and agreement levels.

Conversely, undergraduate and postgraduate dental students, particularly those enrolled in specialized periodontal training, showed relatively high levels of accuracy and inter- and intra-examiner agreement [9,13]. Among undergraduates, a clear trend was observed: diagnostic accuracy increased with the year of study, suggesting that even limited clinical exposure, when supported by structured education, can yield competent performance. Postgraduates and periodontal specialists demonstrated the highest diagnostic accuracy, though paradoxically, expert groups also reported low inter-examiner agreement [9,10]. This may reflect the nuanced and interpretive nature of advanced diagnostic criteria, where more experienced clinicians apply broader clinical judgment.

### 4.2. Diagnostic Challenges Related to Staging and Grading

**Figure 1:**
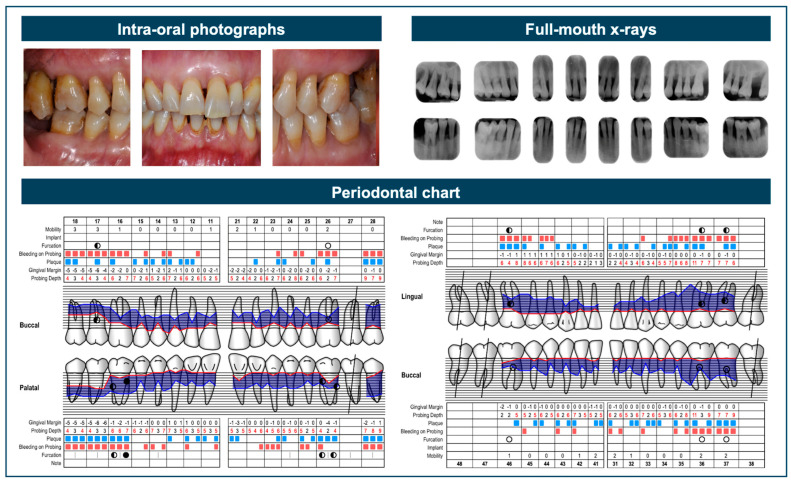
This case represents a diagnostic gray zone, as it shows advanced interdental attachment loss (≥5 mm), which is compatible with both Stage III and Stage IV periodontitis. Key factors to consider include the number of missing teeth, which is fewer than five, although some hopeless teeth may potentially be classified as missing. Additionally, there are complex features typical of Stage III, such as Class II/III furcation involvement, moderate ridge defects, and probing depths ≥6 mm. However, there are also indicators that support case definition as Stage IV, including the need for complex rehabilitation due to flaring and migration of the upper anterior teeth, as well as hypermobility associated with occlusal trauma.

Konrmann and Papapanou [20] were among the first to explore the ‘gray zones’ within the 2018 Classification. The present review demonstrated that clinicians frequently faced significant challenges when implementing the classification in practical clinical settings. One of the most commonly encountered difficulties in clinical classification involves cases that fall on the borderline between Stage III and Stage IV periodontitis. These cases are often misclassified due to subtle distinctions between the two stages. Specifically, when fewer than five teeth have been lost due to periodontitis and the classification must rely primarily on the complexity of treatment needs, clinicians should ask themselves whether the planned management of the patient’s condition will require extensive, multi-disciplinary oral rehabilitation. This decision should be based on a comprehensive evaluation of all potential factors of complexity, rather than a simplistic checklist approach focused on isolated criteria [20,21]. In this regard, clinical experience often allows for greater diagnostic accuracy than what can be achieved through simple algorithms, decision trees, or software applications. Figure 1 illustrates a borderline Stage III/Stage IV periodontitis case.

**Figure 2:**
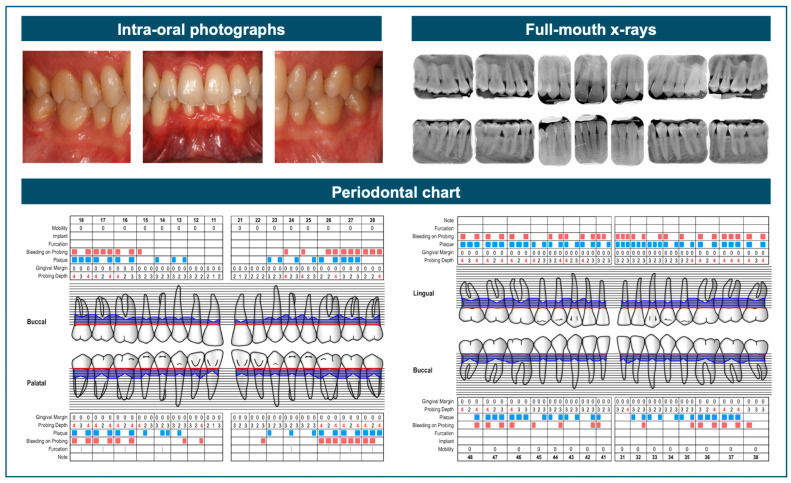
This case falls within a diagnostic gray zone due to the presence of gingival inflammation, characterized by redness, edema, and bleeding on probing, along with sites exhibiting probing depths of 4 mm. Radiographically, there is no evident bone loss reaching one-third of the root length, and only minor qualitative changes in the bone structure can be observed. It is important to distinguish true periodontal pockets from potential pseudo-pockets in order to accurately assess the presence of clinical attachment loss, which in this case would be minimal.

Another recurring diagnostic challenge lies in distinguishing gingivitis from early-stage (Stage I) periodontitis. Stage I is typically defined by minimal attachment loss, often accompanied by early radiographic signs of alveolar bone disruption, such as a break in the integrity of the lamina dura, rather than a marked increase in the distance between the cementoenamel junction and the alveolar crest. Differentiating early periodontitis from gingivitis can be clinically challenging. In ambiguous cases, it is recommended to carry out initial periodontal therapy (Step 1) and re-evaluate probing depths to confirm the presence of true periodontal pockets, as opposed to pseudo-pockets. Figure 2 illustrates a borderline gingivitis/Stage I periodontitis case.

**Figure 3:**
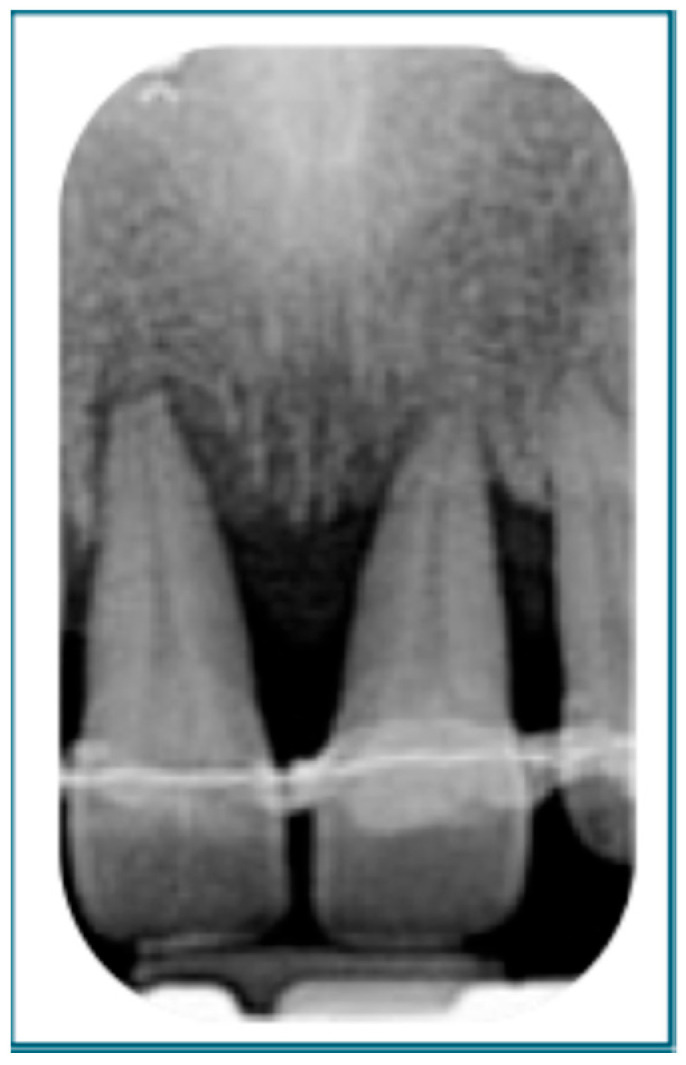
Tooth #11 with hopeless prognosis presenting radiographic bone loss approaching the root apex circumferentially.

Shortly after the introduction of the staging and grading system, several authors highlighted the need to clarify certain points that could potentially mislead clinicians during clinical assessments [22]. Firstly, it was clarified that teeth deemed to have a “hopeless” prognosis should be included in the count of teeth lost due to periodontitis for staging purposes. A tooth has to be considered hopeless when attachment loss extends nearly to the root apex circumferentially, often in combination with Grade III mobility [22]. Figure 3 illustrates a tooth with a hopeless prognosis.

**Figure 4:**
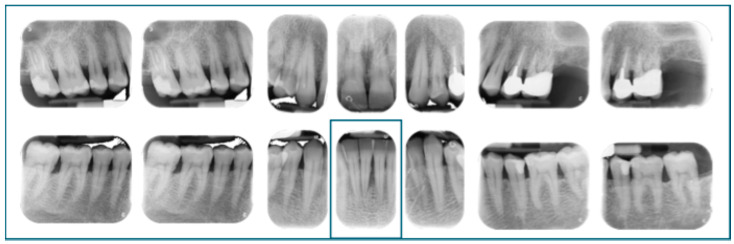
Full-mouth radiographs reveal localized Stage III periodontitis, evidenced by bone loss extending beyond the middle third of the root at tooth 4.1. The narrative summary of this case could also reflects that the remaining dentition is affected by generalized Stage II periodontitis.

Moreover, a common error is to assign extent based on the percentage of teeth affected by periodontitis. Conversely, extent should be based on the percentage of teeth at the stage-defining severity level [9]. However, it is acceptable to describe multiple extents within the same case in the narrative summary, especially when different parts of the dentition exhibit varying severities of disease. For example, a case classified as localized Stage III periodontitis may include regions showing only mild to moderate bone loss [10,20]. Figure 4 illustrates a localized Stage III periodontitis.

**Figure 5:**
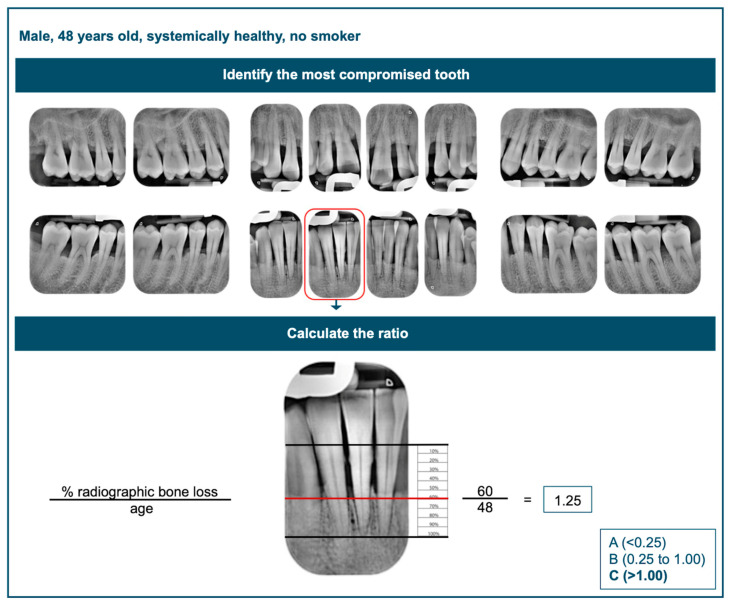
Assignment of grade in absence of longitudinal data and grade modifiers.

Furthermore, in the absence of longitudinal records and key grade modifiers such as smoking or diabetes, grading assessments often become less precise and reliable [9]. Clinicians may struggle to identify the most severely affected tooth and to accurately calculate the percentage of radiographic bone loss in relation to the patient’s age. However, in cases where the patient is a heavy smoker (more than 10 cigarettes per day) or has poorly controlled diabetes (HbA1c > 7.0%), assigning the periodontitis grade becomes significantly more straightforward. Another potential error in assigning the periodontitis grade may arise from not applying the highest relevant criteria. In fact, some clinicians may average the primary criteria and grade modifiers—such as assigning Grade B when the progression rate corresponds to Grade C but no grade modifiers are present, or vice versa—leading to potential misclassification. It should also be noted that in clinical practice, grade A is rarely assigned in the absence of longitudinal data, as the bone loss-to-age ratio required for this diagnosis is relatively low. Figure 5 illustrates the assignment of a grade in the absence of longitudinal data and grade modifiers.

**Figure 6:**
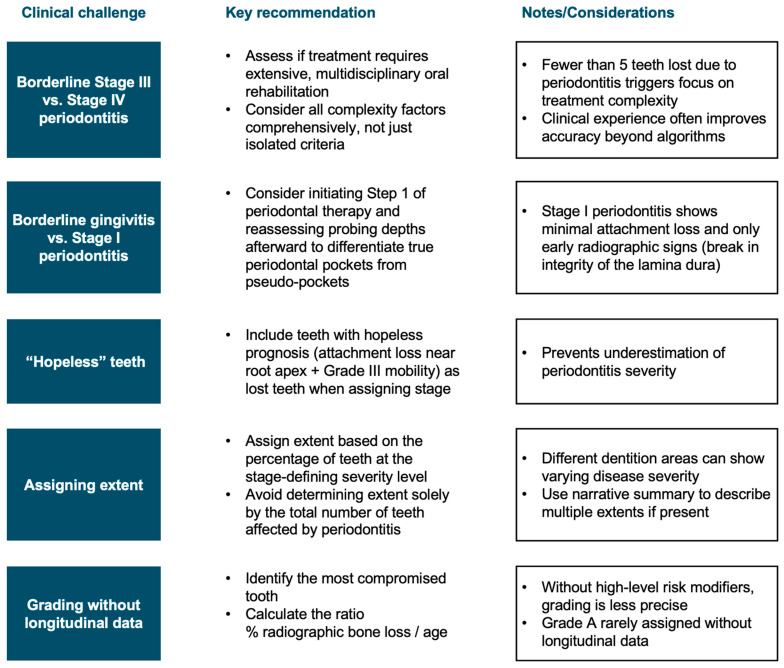
Clinical recommendations to improve staging and grading consistency.

Figure 6 summarizes the main clinical challenges along with practical recommendations to improve diagnostic accuracy.

### 4.3. Staging and Grading Prognostic Value and Role in Treatment

Staging and grading in periodontology are also crucial for predicting disease progression and guiding treatment planning. As outlined in the S3-level EFP guidelines, staging reflects the severity and complexity of periodontitis, guiding decisions on the necessity and type of non-surgical or surgical therapies, as well as the potential need for multidisciplinary management [23,24].

Grading, on the other hand, offers a forecast of disease progression and highlights the influence of modifiable risk factors such as smoking or poor glycemic control. The staging and grading system functions also as a prognostic tool, aiming to predict the likelihood of future tooth loss. However, many clinicians still assess periodontal risk subjectively rather than relying on structured tools, increasing the risk of misclassification. Proper risk assessment tools should serve both diagnostic and educational purposes, helping patients understand their condition. Therefore, inaccuracies in staging and grading can result in suboptimal treatment planning, potentially compromising long-term outcomes. A pivotal study by Ravidà et al. provided the first solid evidence supporting the prognostic value of the staging and grading system [25]. Over a follow-up period of at least 10 years, they found that higher stages and grades were strongly associated with increased tooth loss, regardless of the distribution of the disease. In a subsequent analysis, they confirmed that the combination of a high stage and a high grade significantly elevated the risk of tooth loss. Notably, in patients classified as Stage IV and Grade C, the generalized extent of disease became a significant predictor of future tooth loss [26]. In 2022, Saleh et al. [27] compared the prognostic accuracy of four well-established risk assessment tools: the Periodontal Risk Assessment (PRA) by Lang and Tonetti (2003) [28], the PerioRisk tool from the University of Ferrara (2007) [29], the Periodontal Risk Calculator (PRC) by Page et al. [30], and the staging and grading system. Across 167 patients, all tools demonstrated a good predictive capability, with PerioRisk and PRA outperforming the others, followed by staging and grading. Another recent study also set out to determine two additional outcomes [31]. First, it assessed how many patients required additional non-surgical or surgical interventions during supportive periodontal therapy based on their initial stage and grade. Nearly two-thirds of patients needed further treatment during follow-up. Recurrence risk was strongly linked to a higher stage and grade, as well as poor compliance, the nature of the initial active therapy, smoking, and diabetes. The second objective was to calculate the overall cost of treatment according to the stage, grade, and patient compliance. The results demonstrated that patients with high compliance with maintenance therapy, even those in advanced stages (III/IV) and grades (B/C), experienced lower overall costs in managing disease recurrence. Conversely, for patients with less severe disease (Stage I/II and Grade A), fewer maintenance appointments might offer a cost-effective alternative without compromising outcomes.

### 4.4. Emerging Role of Artificial Intelligence

Advancements in technology have significantly contributed to the emergence and widespread adoption of computer-aided diagnosis in the field of medical imaging analysis. In the early detection of periodontitis, clinicians typically rely on clinical examinations using periodontal probes to assess the tissue around each tooth. However, this procedure is both time-consuming and uncomfortable for patients. As a result, many dental practitioners prefer to use radiographic imaging for the initial screening and diagnosis of periodontal disease, despite its well-known limitations. This has led to a growing need for automated, less invasive diagnostic tools—particularly those powered by artificial intelligence.

In 2021, Cheng and colleagues published one of the first studies aimed at creating an AI-based model to automatically stage periodontitis by estimating bone loss on panoramic radiographs [6]. Since then, as summarized in a recent review, there has been a surge in the development of deep learning models for the classification of periodontitis stages. Most of these studies have focused on panoramic radiographs, with some also using intraoral imaging. A meta-analysis revealed that deep learning-based classification systems offer promising accuracy, suggesting their potential to reduce the workload of dental professionals and improve diagnostic consistency. However, the review also emphasized the need for higher-quality studies to validate these findings [7]. More recently, an innovative hybrid classification framework has been introduced. This system integrates three key components: tooth-level classification, patient-level diagnosis, and a probabilistic model that synthesizes information from multiple prediction sources. By accounting for uncertainty and varying levels of prediction confidence, the model achieved an exceptional diagnostic performance. These results highlight its strong potential for integration into clinical practice as a reliable tool for periodontal assessment [8].

A recent study presented a highly accurate, automated, and non-invasive system developed to digitally measure the gingiva–bone distance and capture detailed information about both soft and hard oral tissues. The platform operates through a comprehensive four-step process: segmentation of intraoral scans, segmentation of cone-beam computed tomography images, fusion of multimodal data, and digital probing for measurement. This technology offers significant potential to enhance clinical workflows in periodontal care by providing a more precise and patient-friendly method for diagnosis and treatment planning [32].

While these technologies are promising, most current studies are preliminary, and further research is required to validate their clinical utility and integration into daily practice.

### 4.5. Strengths and Weaknesses of the 2018 Classification of Periodontitis

The 2018 Classification of Periodontitis has been generally well-received, particularly among less experienced clinicians. Its staging and grading approach aligns well with the objectives and expectations of contemporary periodontology. Furthermore, implementation tools—such as diagnostic flowcharts and digital technologies—are recognized as valuable aids in facilitating its practical application [4,5].

Nonetheless, the ‘gray zones’ within the classification remained challenging even for experienced periodontists, with some reporting a perceived subjectivity in their diagnostic decisions. Moreover, although not explicitly examined in the reviewed studies, an important consideration is that the system’s dependence on comprehensive medical histories, full-mouth clinical assessments, and radiographic evaluations adds complexity to its application in epidemiological research settings [33]. For the above-mentioned reasons, it is critical that clinical and epidemiological studies provide detailed descriptions of examiner training and calibration procedures to ensure the reliability of the data collected.

### 4.6. Limitations

A key limitation of this review is its narrative nature, which lacks the methodological rigor and reproducibility of a systematic review. Conversely, West et al. [33] conducted a systematic review that offered a more structured assessment of the clinical application of the 2018 AAP/EFP Classification of Periodontal Diseases. However, the narrative approach adopted here allowed for a broader and more interpretive exploration of the literature, emphasizing contextual factors, examiner-related variability, and practical implementation issues that may not emerge through more narrowly focused methodologies.

## 5. Conclusions

Diagnostic accuracy and consistency in applying the 2018 Classification of Periodontitis has been variable. Examiner performance is strongly influenced by education, experience, and the use of diagnostic aids. Studies consistently highlight the need for standardized training, and implementation tools to optimize the use of the classification.

## Figures and Tables

**Table 1:** Characteristics of studies addressing diagnostic accuracy, as well as inter- and intra-examiner agreement.

Authors and Year	Country	Examiners: Number and Education	Gold-Standard Diagnosis	Additional Lecture on 2018 Classification	Implementation Tools	No. of Cases	Periodontal Status
Marini et al., 2021 [9]	Italy	30 total: 10 periodontal experts, 10 general dentists, 10 final-year students	1 periodontal expert (involved in development of classification)	Detailed instructions and training on 3 cases (not in the study)	No	25	Stage I–IV periodontitis
Ravidà et al., 2021 [10]	USA	103 periodontal experts	5 periodontal experts (involved in development of classification)	No	No	9	Stage II–IV periodontitis
Oh et al., 2021 [11]	USA	64 total: 31 periodontal faculty/postgraduate students, 33 non-periodontal postgraduate students	3 periodontal experts	No	Questionnaire with closed and open-ended questions	3	Stage II–IV periodontitis
Abou-Array et al., 2021 [12]	USA	131 total: 57 second-year students, 45 fourth-year students, 17 ortho postgraduate students (OS), 12 perio postgraduate students (PS)	2 periodontal experts	1 lecture one month before examination	No	10	Health (normal/ reduced periodontium), gingivitis, Stage I–IV periodontitis
Abrahamian et al., 2022 [13]	Spain	174 periodontal experts and postgraduate students	7 internationally recognized periodontal experts	No	Algorithm by Tonetti and Sanz (2019) [4]	5	Stage III–IV periodontitis
Pakdeesettakul et al., 2022 [14]	Thailand	152 total: periodontal experts, postgraduate students, fifth-year students	6 periodontal experts	No	Consensus report (Group A); simplified flowchart (Group B)	25	Health, gingivitis, Stage I–IV periodontitis
Bumm et al., 2023 [15]	Germany	83 dental students: 43 w/o experience, 40 with experience	Consensus of the investigators	2 regular 45 min lectures; test group had extra 45 min on Tonetti and Sanz algorithm	Algorithm by Tonetti and Sanz (2019) [4] for Group B only	2	Stage III periodontitis
Marini et al., 2024 [5]	Italy	10 general dentists	1 periodontal expert (involved in development of classification)	Detailed instructions and training on 3 cases (not in the study)	Dedicated software developed by the Italian Society of Periodontology and Implantology	25	Stage I–IV periodontitis
Roshdy Abdelrasoul et al., 2024 [16]	Saudi Arabia	52 senior-year dental students	2 periodontal experts	Introductive seminar (2018 vs. 1999), discussion, pre- and post-tests	No	12	Stage I–IV periodontitis
Raza et al., 2024 [17]	USA	1 periodontal expert	3 periodontal experts	No	No	336	Gingivitis, Stage I–IV periodontitis
Alshehari et al., 2024 [18]	Saudi Arabia	52 fourth- and fifth-year dental students	1 periodontal expert	Course during third year	No	7 (only 1 sextant presented)	Stage I–IV periodontitis

**Table 2:** Diagnostic agreement with gold-standard diagnosis.

Authors and Year	Agreement with Gold Standard (% or OR)			
	Stage	Extent	Grade	Overall
Marini et al., 2021 [9]	UG: 81.6%/GD: 64.4%/PE: 82%	UG: 87.6%/GD: 76.4%/PE: 84%	UG: 74.4%/GD: 67.6%/PE: 72.4%	UG: 53.6%/GD: 37.6%/PE: 50.4%
Ravidà et al., 2021 [10]	76.6%	84.8%	82%	-
Oh et al., 2021 [11]	Case 1: P 52% / NP 48% Case 2: P 68%/ NP 64% Case 3: P 94%/ NP 73%	-	Case 1: P 72%/ NP 42% Case 2: P 81% / NP 73% Case 3: P 39% / NP 33%	-
Abou-Array et al., 2021 [12]	-	-	-	-
Abrahamian et al., 2022 [13]	UF: 71.4%/SC: 65.6%/PG: 68.2%/Total: 68.7%	UF: 76.1%/SC: 70.0%/PG: 77.7% / Total: 75.5%	UF: 80.8%/SC: 86.1%/PG: 82.3%/Total: 82.4%	-
Pakdeesettakul et al., 2022 [14]	-	-	-	Flowcharts: 88.21% No flowcharts: 87.26%
Bumm et al., 2023 [15]	Experience: OR 3.704/Algorithm: OR 4.425	Experience: OR 1.664/Algorithm: OR 1.767	Experience: OR 6.993/Algorithm: OR 30.303	Experience: OR 4.132/Algorithm: OR 11.905
Marini et al., 2024 [5]	74.4%	82.8%	84.0%	53.6%
Roshdy Abdelrasoul et al., 2024 [16]	-	-	-	Pre-test: 63.8% ± 14.8%/Post-test: 75.6% ± 12.7%
Raza et al., 2024 [17]	90%	100%	100%	-
Alshehri et al., 2024 [18]	Fourth year: 56.57%/fifth year: 59.79%	-	Fourth year: 68.57%/fifth year: 75.6%	-

UF = university faculty; SC = specialist clinicians; UG = undergraduates; GD = general dentists; PE = periodontal experts; P = periodontal background; PG = postgraduate students; NP = non-periodontal background.

**Table 3:** Inter- and intra-examiner agreement across examiners.

Authors and Year	Inter-examiner Agreement [kappa (k) or %]				Intra-examiner Agreement [kappa (k), ICC or %]			
	Stage	Extent	Grade	Overall	Stage	Extent	Grade	Overall
Marini et al., 2021 [9]	UG: k = 0.65 / GD: k = 0.36 / PE: k = 0.58	UG: k = 0.64 / GD: k = 0.31 / PE: k = 0.36	UG: k = 0.52 / GD: k = 0.44 / PE: k = 0.42	-	UG: k = 0.95 / GD: k = 0.92 / PE: k = 0.82	UG: k = 0.98 / GD: k = 0.79 / PE: k = 0.88	UG: k = 0.88 / GD: k = 0.86 / PE: k = 0.87	-
Ravidà et al., 2021 [10]	k = 0.49	k = 0.51	k = 0.50	k = 0.48	-	-	-	-
Oh et al., 2021 [11]	-	-	-	P: k = 0.41 / NP: k = 0.28 / Total: k = 0.34	-	-	-	Total: k = 0.34
Abou-Array et al., 2021 [12]	-	-	-	-	-	-	-	D2: k = 0.24 / D4: k = 0.26 / OS: k = 0.30 / PS: k = 0.55/Total: k = 0.24
Abrahamian et al., 2022 [13]	68.7%	75.5%	82.4%	-	k = 0.71 (82.3%)	k = 0.52 (83%)	k = 0.85 (91.4%)	-
Pakdeesettakul et al., 2022 [14]	-	-	-	-	-	-	-	Flowcharts: 58.26%/No flowcharts: 55.84%
Bumm et al., 2023 [15]	-	-	-	-	Experience: 52.4%/Algorithm: 80.5%	Experience: 73.8%/Algorithm: 82.9%	Experience: 50.0%/Algorithm: 95.1%	-
Marini et al., 2024 [5]	k = 0.81	k = 0.60	k = 0.63	-	-	-	-	-
Roshdy Abdelrasoul et al., 2024 [16]	-	-	-	Pre-test: k = 0.215 Post-test: k = 0.427	-	-	-	-
Raza et al., 2024 [17]	-	-	-	-	-	-	-	-
Alshehri et al., 2024 [18]	-	-	-	-	-	-	-	-

UG = undergraduates; GD = general dentists; PE = periodontal experts; P = periodontal background; NP = non-periodontal background; D2 = second-year undergraduate students; D4 = fourth-year undergraduate students; OS = ortho postgraduate students; PS = perio postgraduate students.

**Table 4:** Factors affecting staging and grading.

Authors and Year	Factors Affecting Staging	Factors Affecting Grading	Other Findings
Marini et al., 2021 [9]	- Stage most often overestimated - Borderline cases less consistently and accurately diagnosed - Extent often underestimated	- Grade often underestimated - Bone loss/age associated with less consistency and accuracy	- General dentists performed less well - Staging easier than grading and extent - Almost perfect consistency over time - Moderate consistency across examiners
Ravidà et al., 2021 [10]	- Stage severity based on interdental CAL; only CAL attributable to periodontitis should be used - Hopeless teeth included in teeth lost count - Stages III and IV share essential identifiers; Stage IV needs added-complexity factors - Stage I or II cases cannot be upshifted based only on complexity - Generalized extent only if >30% of teeth show defining characteristics	-	-
Oh et al., 2021 [11]	- Fair to moderate agreement - Non-periodontists badly accustomed to 2018 classification	- Grading more difficult without previous records; main parameter: bone loss/age ratio	- 74% of periodontal cohort used 2018 classification exclusively - 79% of non-periodontal cohort unaware or not using the 2018 classification
Abou-Array et al., 2021 [12]	- Stage often overdiagnosed - Difficulties distinguishing Stage I and differentiating Stages III and IV - Localized periodontitis often underdiagnosed	-	- Tendency to prioritize stage over grade and extent
Abrahamian et al., 2022 [13]	- Difficulty distinguishing stage III and IV due to hopeless teeth and complexity factors like masticatory dysfunction - Extent assessment should follow stage determination	Grade is easiest to assess	- New classification successfully diagnoses periodontitis cases with high concordance - Easier to correctly assign grade > extent > stage - Neither current position nor experience influenced outcomes
Pakdeesettakul et al., 2022 [14]	-	-	- Flowcharts improved clinician confidence - Most diagnostic errors were minor details, especially in periodontitis cases
Bumm et al., 2023 [15]	- Experience and algorithm usage influence staging - Sole use of CAL may underestimate loss in previously treated patients - Assessment of extent not influenced by experience/algorithm	- Experience and algorithm usage influence grading	- Algorithms may aid implementation of current classification among operators with different experience levels
Marini et al., 2024 [5]	- Stage overestimation may occur because only a single site meeting a specific criterion (e.g., one probing depth >6 mm) is sufficient to escalate the diagnosis from Stage II to Stage III - Extent overestimation often describes the overall periodontitis distribution rather than accurately defining the stage	- Grade overestimation is frequently linked to clinical phenotype evaluation, where the degree of tissue destruction relative to the amount of plaque has a significant impact on grading	- General dentists could benefit from digital support tools to assist in accurately assigning stage and grade - Failure to achieve a correct diagnosis could be primarily attributed to improper data entry - Staging and grading should be approached as a critical diagnostic process rather than merely a “box-checking” task
Roshdy Abdelrasoul et al., 2024 [16]	- Insufficient training in 2018 classification (dominant 1999 classification also among teachers) - COVID-19 influenced insufficient clinical practice - Difficulty in differentiating stage III and IV	-	- The training process has the potential to enhance inter-examiner agreement compared with the gold standard
Raza et al., 2024 [17]	- Number of teeth lost without previous records can increase stage	- Differentiation between bone loss and bone remodeling for grading	- Practical to retroactively diagnose patients previously diagnosed with 1999 AAP/CDC classification using the 2017 AAP/EFP system
Alshehari et al., 2024 [18]	- One sextant evaluation influenced staging due to limited clinical experience of undergraduates	- One sextant evaluation influenced grading similarly	- High inter-examiner agreement among fourth- and fifth-year undergraduates using the 2018 classification

## Data Availability

The original contributions presented in this study are included in the article. Further inquiries can be directed to the corresponding author.
